# Biobanking of human gut organoids for translational research

**DOI:** 10.1038/s12276-021-00606-x

**Published:** 2021-10-18

**Authors:** Francesca Perrone, Matthias Zilbauer

**Affiliations:** 1 Department of Paediatrics, University of Cambridge, Addenbrooke’s Hospital, Cambridge, UK; 2 Department of Paediatric Gastroenterology, Hepatology and Nutrition, Cambridge University Hospitals, Addenbrooke’s, Cambridge, UK; 3 Wellcome Trust-Medical Research Council Stem Cell Institute, University of Cambridge, Cambridge, UK

**Keywords:** Translational research, Intestinal stem cells

## Abstract

The development of human organoid culture models has led to unprecedented opportunities to generate self-organizing, three-dimensional miniature organs that closely mimic in vivo conditions. The ability to expand, culture, and bank such organoids now provide researchers with the opportunity to generate next-generation living biobanks, which will substantially contribute to translational research in a wide range of areas, including drug discovery and testing, regenerative medicine as well as the development of a personalized treatment approach. However, compared to traditional tissue repositories, the generation of a living organoid biobank requires a much higher level of coordination, additional resources, and scientific expertise. In this short review, we discuss the opportunities and challenges associated with the generation of a living organoid biobank. Focusing on human intestinal organoids, we highlight some of the key aspects that need to be considered and provide an outlook for future development in this exciting field.

## Tissue banks: What to consider when biobanking mini-guts

Repositories of mini-organs grown from patient-derived stem cells to closely resemble the architecture and function of the intestinal tract, provide an invaluable resource for drug discovery, regenerative medicine and personalized therapeutics. However, the construction and maintenance of such biobanks involve many scientific, logistic and ethical complexities that merit careful attention from both researchers and clinicians. Francesca Perrone and Matthias Zilbauer from the University of Cambridge, UK, review the various human intestinal organoid collections in existence, many of which focus on particular diseases such as colorectal cancer or cystic fibrosis. The authors discuss best practices for generating and storing these biological resources, emphasizing the need to include relevant patient information while protecting the identity of tissue donors. The article focuses on gut organoids, but should be relevant to any organoid biobanking effort.

## Introduction

A vast amount of human tissue is being collected for routine clinical purposes on a daily basis, and often, only a fraction of the available material is required. It has long been recognized that the prospective storage of such excess tissue in the form of biobanks or tissue banks provides extensive opportunities for future research studies^1–6^. Indeed, many ground-breaking discoveries in both basic and translational biomedical research have benefitted from the analyses of human tissue stored in large biobanks. Importantly, the value of such tissue banks is directly associated with the quality of their organization, which includes the detailed documentation of patient information as well as sample processing and storage. Major progress has been made in this field, leading to the development of large, highly sophisticated biobanks^7–10^. However, a major limitation of stored tissue is the inability to perform functional experiments, which are required for many areas, particularly in translational research. The development of human organoid culture models has provided researchers with unprecedented opportunities to generate miniature organs that closely mimic in vivo anatomy and pathophysiology and retain patient-specific characteristics^11^. To date, organoids have been successfully generated from a wide range of human tissues, including the entire intestinal tract^12–17^. Indeed, since the development of the first organoid culture model from the mouse small intestine approximately one decade ago^18^, substantial progress has been made in this area^19–21^. Although the majority of topics discussed in this review will equally apply to any organoid biobank, we will focus on the human intestine based on our own experience in this field.

**Fig. 1:**
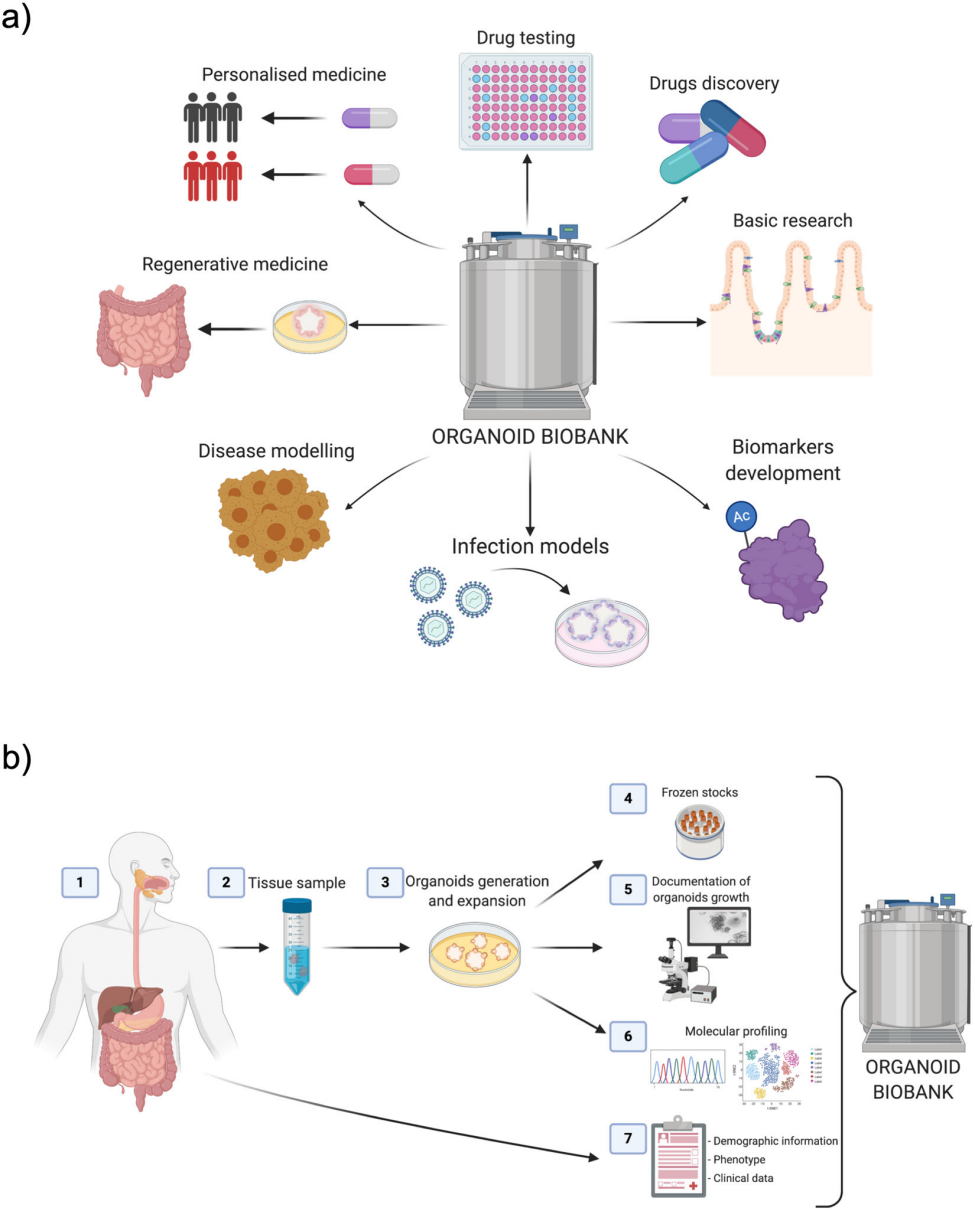
Establishment of an organoid biobank and its applications. **a** Applications for next-generation organoid biobanks in translational research. **b** Basic workflow for the generation of an organoid biobank. Key areas requiring careful consideration and detailed documentation include (1) donor information, (2) tissue of origin, (3) culture methodology and passage number, (4) generation of frozen stocks, (5) documentation of organoid growth and morphology, (6) molecular profiling of organoids and/or primary tissue, and (7) relevant clinical information. All images were created with BioRender.com.

Intestinal organoids are defined as self-organizing three-dimensional structures that closely mimic the in vivo situation. In principle, they can be generated either from pluripotent stem cells (e.g., induced pluripotent stem cells (iPSCs) or embryonic stem cells (ESCs)) or adult stem cells^22–24^. The latter is located in the crypts of the intestinal epithelium and, upon provision of the appropriate scaffold and culture conditions that mimic the stem cell niche, will self-organize into mature intestinal epithelial organoids (IEOs), which contain all epithelial cell subsets structured in a crypt villus axis. To date, IEOs have been successfully generated from all gut segments of the human intestinal tract, and several studies have shown that organoids display gut segment-specific features in vitro^25–27^. Furthermore, increasing evidence suggests that patient-derived IEOs retain donor-specific properties such as age, gender, and disease-associated differences^9,28,29^. As a result, IEOs provide scientists with unprecedented opportunities to use these models as highly versatile translational research tools. Areas of clinical relevance include but are by no means limited to the development of novel therapeutics, testing of existing drugs with an aim to personalize treatment based on responses as well as the use in regenerative medicine (Fig. 1a)^11,30–32^.

Importantly, IEOs can be expanded in vitro over prolonged time periods and frozen stocks can be generated at any time during this process. This phenomenon allows the generation of living biobanks and biorepositories that can be shared with researchers across the globe and in particular those who do not have access to human tissue samples (Fig. 1b). However, despite the extraordinary progress in the field of human IEOs over the last decade, in regard to translating science into patient care, additional caution and stringent quality control procedures are critical first steps. In the following, we will briefly summarize examples of existing human organoid biobanks and highlight considerations for generating and distributing IEOs as part of a biobank or biorepository. Finally, we will provide an outlook on future developments and key strategic goals for the field. We will primarily focus on mucosa-derived IEOs, as our group has extensive expertise in this field. Nevertheless, most aspects we discuss will also apply to iPSC-derived gut organoids, and we therefore will not distinguish between them unless otherwise specified.

## Existing human intestinal organoid biobanks

The major value of generating organoid biobanks for translational research purposes has long been recognized and is reflected in the rapidly increasing number of studies reporting on the successful establishment of large patient-derived, living biobanks. Interestingly, the vast majority of such sample collections have been derived from patients diagnosed with various malignancies, including those affecting the gastrointestinal tract. Examples include colorectal cancer, metastatic colorectal and esophageal cancer, gastric cancer, pancreatic cancer, and advanced rectal cancer (summarized in Table 1)^9,28,29,33–38^. In all of these cases, the generation of patient-derived biobanks included a detailed description of clinical phenotype and patient records, characteristics of samples used to generate organoids (such as macroscopic and/or histological appearance), as well as molecular profiling of both the tissue of origin and/or the resulting organoid cultures. Furthermore, most studies also provide evidence for generated organoids to recapitulate patient and disease-specific pathophysiological processes, thereby validating them as translational research tools^9,28,29^. Importantly, several studies have highlighted the ability of generated organoids to personalize treatment by demonstrating a patient-specific response to existing treatments^34,35^.

**Table 1:** Overview of existing human intestinal organoid biobanks.

Organ of origin	Disease	Number of lines	Molecular/phenotypic profiling analysis	Academic/ Commercial	References
Colorectum	Colorectal carcinoma	22	Whole-exome sequencing, gene expression	Academic	^28^
Colorectum	Colorectal carcinoma	55	Whole-exome sequencing, gene expression, immunohistochemistry	Academic	^29^
Pancreas	Pancreatic ductal adenocarcinomas	39	Whole-exome sequencing, methylation, gene expression, immunohistochemistry and in situ hybridization	Academic	^33^
Pancreas	Pancreatic ductal adenocarcinomas	114	Whole-genome sequencing, whole-exome sequencing, gene expression	Academic	^34^
Colorectum and esophagus	Colorectal and gastroesophageal cancer	Not specified	Whole-genome sequencing, gene expression, immunohistochemistry, CISH, and FISH	Academic	^9^
Stomach	Gastric cancer	63	Whole-genome sequencing, gene expression, CISH, gene set enrichment analysis	Academic	^35^
Intestine	Cystic fibrosis	664	Whole-genome sequencing	Academic	^36^
Pancreas	Pancreatic cancer	30	Whole-genome sequencing, gene expression, immunohistochemistry	Academic	^37^
Stomach	Gastric cancer	20	Whole-genome sequencing, gene expression	Academic	^38^
Intestine, lungs, colorectum, head, neck, liver, ovary	Normal, cancer, genetic diseases	>1000	Not specified	Commercial	https://huborganoids.nl/
Intestine, brain, lungs, muscle, breast, kidney, skin	Cancer	Not specified	Not specified	Commercial	www.attc.org
Colorectum	Cancer	Not specified	Whole-genome sequencing	Commercial	(https://cellesce.com)
Intestine	iPSC-derived intestinal organoids	Not specified	Gene expression	Commercial	https://www.definigen.com/products/intestinal/organoid/
Retina, gut, brain, pancreas, Inner ear, liver	Stem cells derived organoids	Not specified	Not specified	Commercial	https://sunbioscience.ch/
Colon	iPSC-derived colon organoids	Not specified	Gene expression analysis of colon markers, genotyping by STR analysis	Commercial	https://www.sigmaaldrich.com/

In contrast to reports on malignancies, only a limited number of studies have been able to demonstrate the value of living organoid biobanks for other GI-related conditions. This phenomenon is in part due to the presence of genetic alterations in malignant tissue which are faithfully retained in organoid cultures and impact cellular function. Indeed, cystic fibrosis (CF) represents another example of a genetic, nonmalignant condition for which the value of patient-derived organoids as translational tools to stratify treatment has been demonstrated. CF is caused by various mutations in the CF transmembrane conductance regulator (CFTR) gene^39^. Human IEOs derived from rectal biopsies of CF patients have shown a strong correlation with in vivo drug responses by using a forskolin-induced swelling assay in vitro^40^. Based on these discoveries, a living organoid biobank was generated representing over 600 patient-derived organoid cultures^36^. Notably, all generated organoid lines were shown to retain patient-specific mutations in the CFTR gene, some of which could be corrected using SpCas9-induced DNA template recombination or SpCas9-adenine base editing^36,41^. The latter fact further highlights the use of genome editing to further enhance the value of organoid cultures as translational research tools.

One of the most extensive organoid biobanks was established by the The Hubrecht Institute, the University Medical Center Utrecht, and the Royal Netherlands Academy of Arts and Sciences (KNAW) as part of Hubrecht Organoid Technology (HUB, https://huborganoids.nl/). The collection contains more than 1000 organoid lines generated from different organs and diseases, including breast, colon, head, neck, intestine, liver, lung, ovarian, and pancreatic tumors, as well as a large number of intestinal organoids derived from CF patients. Genetic and histological data of all lines are available along with detailed information about the sensitivity to specific drugs for treatment strategies in the case of CF patient-derived organoids.

In addition to academic institutions, organoid biobanks have been established by commercial entities such as Sigma-Aldrich, the American Type Culture Collection (ATCC, (www.attc.org)), Cellesce (https://cellesce.com) and DefiniGEM (https://www.definigen.com/products/intestinal/organoid/). These collections offer a wide range of human organoids derived from either iPSCs or primary tissues (Table 1) covering both healthy donors and patients with established diseases. Detailed information on both generated organoid cultures and donors is also available and includes molecular profiling, the results of drug screening, and the presence of specific genetic mutations in some instances. A proportion of organoid lines available via these biobanks were generated from iPSCs, further extending the experimental repertoire and potential applications of cultures available to researchers.

In summary, the number of organoid biobanks and repositories has been increasing in recent years, with a large proportion of disease-related collections focused on malignant and/or genetically well-defined conditions.

## Important features of organoid biobanks

Despite major progress made in this field and the high quality of human organoid biobanks established to date, several areas require consideration to further improve the value of living organoid biobanks in the future. In the following, we will summarize some of the key aspects that are critical for the generation of a living organoid biobank to enhance their value for translational research.

### Patient and sample details

One of the most important advantages of patient-derived organoids is the fact that these cultures retain the molecular as well as functional characteristics of their donors^26,28^. Hence, providing detailed donor information directly correlates with the utility of generated organoids. In addition to basic characteristics such as gender, age, ethnic background, and potential disease, additional information such as medication, diet, family history of any medical conditions, or the presence of gastrointestinal symptoms are all highly relevant to a wide range of possible research questions. Furthermore, prospective documentation of disease outcome and treatment history, including response to individual treatments, will allow the correlation of organoid-derived molecular signatures or potential functional differences with clinical phenotype. In other words, the documentation of patient details forms the basis for the use of organoids as tools to develop personalized treatment approaches and/or clinical biomarkers (Fig. 1b).

Similarly, the type of tissue samples used to generate IEOs is also critically important. The tissue samples used vary from mucosal biopsies (obtained during routine clinically indicated endoscopy) and surgical resection material to deceased tissue donation. Each of these tissue types may impact the cellular function of generated organoids even if samples were obtained from the same organ/gut segment. In addition, the time from obtaining tissue to initiating organoid cultures, including specification about possible tissue storage, may also impact downstream analyses and should therefore be recorded. Finally, the macroscopic and histological appearance of the intestinal mucosa from which the tissue sample was taken provides highly valuable information on the in vivo microenvironment at the time of sampling and is therefore relevant for the interpretation of experimental results obtained from generated organoids. Providing representative histological images (e.g., hematoxylin and eosin stains) of the original tissue used to generate organoids further enhances the value of the generated lines for future applications.

We would like to highlight the fact that there is currently a lack of published evidence to support the possible impact of the factors mentioned above on generated organoids. However, based on our experience and unpublished data, we believe that these factors are indeed critically important, and unfortunately, many existing studies lack the required information. Hence, prospective, detailed documentation will help to address these important issues in the future and aid the interpretation of generated data as part of published work.

In summary, the value and future use of an organoid biobank for basic and translational research studies critically depend on the information provided on donor and tissue samples used to generate cultures. Despite the major complexities and additional work required to record such details, the increase in value will no doubt outweigh the invested resources.

### Culture conditions and documentation of growth

The initiation and long-term culture of human IEOs were first described by Sato et al.^15^ just over one decade ago. Since then, major progress has been made with regard to optimizing existing protocols. Although the basic culture conditions used are largely comparable between studies and research groups, even subtle variations could lead to substantial differences in organoid phenotype, growth or any downstream analyses performed. One example of such variation is the source of Wnt3a, which is a key component of human IEOs culture medium^42,43^. Although Wnt3a is commercially available as a recombinant protein, its use in the culture of human gut organoids has been reported to be insufficient, as it results in reduced organoids growth^44^. As a result, many studies report the use of conditioned medium-producing cell lines as a source of Wnt3a^45,46^. However, although this approach appears to be superior to the use of recombinant protein, based on our experience, there is substantial variation in batches of conditioned medium, which may impact on organoid growth and/or molecular processes. More recently, a new next-generation surrogate Wnt ligand able to bind both the Wnt receptor (Frizzled) and coreceptors (Lrp5/6) was developed and shown to support the long-term growth of human IEOs^47^. The use of this next-generation recombinant Wnt surrogate will likely improve the reproducibility of organoid work as information on specific concentrations used can be provided. Another example of possible variation in organoid culture comes with the use of different scaffolding reagents to support culture growth. Matrigel, derived from mouse sarcoma, is among the most frequently used extracellular matrix scaffolds and has been shown to adequately support human gut organoid growth and 3D self-organization in many studies^48,49^. However, the composition of Matrigel remains ill-defined and appears to display substantial batch variation, which could impact on reproducibility and comparability of studies^50,51^. Alternative options include animal-derived gel matrixes such as collagen type I and the more recently developed synthetic scaffolds using artificial hydrogels such as polyethylene glycol (PEG), which showed comparable results to those of Matrigel in supporting organoids growth^52,53^. The latter benefits from a clearly defined composition and adjustable mechanical properties, which may reduce the risk of technical variation among studies^54,55^. These examples highlight the need for organoid repositories to provide detailed documentation of culture conditions used by paying particular attention to detail, as minor changes can lead to substantial differences in the results generated.

Regardless of the culture conditions used, documenting organoid growth and structure is critically important and highly valuable. This process can be performed using light microscopy at key stages, including budding stages following the first initiation of cultures, immediately after subculturing, and before frozen stocks are being generated. Information on how many organoids are frozen is also helpful for downstream users and should be considered as standard practice. Similarly, information is routinely provided when sharing cell lines, such as details on regular testing of tissue culture medium for contaminants^56,57^.

The number of passages and culture duration are also critically important and should therefore be recorded. Although human IEOs can be kept in culture for months, even years, the potential impact on cellular phenotype remains largely unknown. Indeed, it is highly likely that artificial culture conditions and the lack of certain external stimuli present in vivo (e.g., cell-cell signaling, microbiota, exposure to nutrients, etc.) may impact epithelial cell function and/or alter molecular profiles. Hence, the generation of robust evidence on the impact of culture duration on human organoids with regard to phenotypic changes (both transient and/or permanent) should be considered a priority in the field. Moreover, documentation of culture duration and passage number must form an essential part of the information provided (Fig. 1b).

### Cryopreservation of tissue for organoid generation

The ability to generate organoids from cryopreserved human tissue has substantially increased the value of samples obtained both for routine clinical care and for specific research settings. A number of protocols have been published that allow cryopreservation of tissue samples followed by successful establishment of organoids even after long-term storage^58–60^. In principle, these protocols require tissue samples to be transferred to freezing medium followed by a gradual reduction in temperature down to −80 °C and transfer to liquid nitrogen storage^59,61^. Success rates for the generation of organoids from frozen tissue are likely to be lower than those for fresh tissue; however, based on our own experience, up to 80% of adequately cryopreserved gut biopsy samples will give rise to viable organoids lines^59,62^. Importantly, these methods will further broaden the application of human organoid technologies and the opportunity for the generation of large living biobanks, as samples can be obtained from patients treated at smaller hospitals that do not have the required research infrastructure to initiate organoid cultures themselves.

However, although based on our experience, organoids generated from frozen tissue samples appear to display the same growth characteristics and morphology as cultures derived from fresh tissue, a freeze-thaw cycle and long-term storage could impact on the cellular function of the generated organoid cultures. Further studies are needed to address this important issue. Thus, detailed information on freezing methods and storage duration should be routinely recorded.

### Molecular profiling of human IEOs

In addition to providing detailed information on donors, tissues, and organoid culture, generating molecular profiles of stored IEOs and/or the primary tissue used to generate these cultures can vastly increase their future value for translational research studies (Fig. 1b). As outlined above, numerous studies have demonstrated the application of organoids in translational research studies by linking organoid function to molecular profiles, mainly genetic profiles (Table 1)^34,63–65^. However, in contrast to the use of genotype to subclassify organoids and stratify possible treatments, other molecular profiles are more likely to change during in vitro culture, making it more challenging to identify a potential correlation with disease, phenotype or response to treatment. For example, transcriptional profiles generated from human IEOs will vastly vary according to a number of factors, such as culture duration following initiation or splitting, the level of differentiation (i.e., profiled in maintenance medium versus in vitro differentiation) in culture medium, and scaffolds and growth factors used. Other molecular signatures/mechanisms, such as DNA methylation, might also be useful, as they have been shown to be highly stable in culture and retain both gut segment, age, and disease-specific alterations in human IEOs^25,66^. Future studies will be able to elucidate these issues further and help to streamline the molecular profiling of organoid biobanks in the future. Currently, the provision of genotypes derived from the original tissue used to generate organoids will substantially increase their future use.

## Ethical considerations

Although next-generation living organoid biobanks have substantial potential and provide unprecedented opportunities for future translational research, they raise numerous ethical issues beyond those that apply to sharing primary human tissue samples. Developments in this important area are ongoing, and due to the complexity of the subject, providing further details is beyond the scope of this review article. However, we refer the interested reader to a number of excellent articles on this topic which highlight some of the key issues including the difficulties in balancing maximizing the benefit of organoid culture technology by making them widely available and providing detailed accompanying information whilst at the same time protecting donor identity^6,67–71^. While close collaboration with pharmaceutical companies forms a vital step in the process of translating any academic discoveries from the bench to the bedside, the prospect of profit-making from human tissue donated for academic research is ethically contentious and likely to raise concern among the wider public^72^.

These are only some of the major ethical issues that require urgent and careful attention to ensure the timely development of sound guidelines for scientists, clinicians, and other stakeholders.

## Future directions and conclusions

The storage and subsequent sharing of human organoid cultures as part of next-generation biobanks have the potential to substantially increase their value and provide major opportunities for basic and translational research. However, the complexities involved in both generating and culturing human organoids as well as storing not only the tissue but also relevant information provide significant additional challenges. Furthermore, given that organoid models are being rapidly developed and improved, validation and quality control studies are urgently needed to inform best practice and support the development of widely accepted protocols. Key areas include the potential impact of long-term in vitro culturing on molecular signatures and cellular function as well as more detailed validation studies aiming to determine the degree to which organoids retain disease-specific features in culture. The potential impact of long-term storage and/or freeze-thaw cycles as fundamental aspects of biobanking must be further explored. Finally, the establishment of clear ethical guidelines that cover areas such as commercialization, patient consent, and the use of organoids for future, non-specified research are needed to ensure the will and privacy of donors are protected.

In conclusion, despite major challenges associated with the generation of next-generation living organoid biobanks, their future use in translational biomedical research is likely to contribute to many groundbreaking discoveries. A collaborative and well-organized effort from the scientific community is required to ensure timely developments in this exciting area.
